# Exogenous calcium: Its mechanisms and research advances involved in plant stress tolerance

**DOI:** 10.3389/fpls.2023.1143963

**Published:** 2023-03-21

**Authors:** Di Feng, Xuejie Wang, Junping Gao, Chenxi Zhang, Hao Liu, Ping Liu, Xiaoan Sun

**Affiliations:** ^1^ Shandong Facility Horticulture Bioengineering Research Center, Weifang University of Science and Technology, Shouguang, Shandong, China; ^2^ Key Laboratory of Crop Water Requirement and Regulation of the Ministry of Agriculture and Rural Afairs/Farmland Irrigation Research Institute, Chinese Academy of Agricultural Sciences, Xinxiang, Henan, China

**Keywords:** abiotic stresses, exogenous calcium, Ca ^2+^, plant tolerance, plant respiratory metabolism

## Abstract

Abiotic stresses are various environmental factors that inhibit a normal plant growth and limit the crop productivity. Plant scientists have been attempting for a long time to understand how plants respond to these stresses and find an effective and feasible solution in mitigating their adverse impacts. Exogenous calcium ion as an essential element for the plant growth, development and reproduction has proven to be effective in alleviating plant stresses through enhancing its resistance or tolerance against them. With a comprehensive review of most recent advances and the analysis by VOSviewer in the researches on this focus of “exogenous calcium” and “stress” for last decade, this paper summarizes the mechanisms of exogenous calcium that are involved in plant defensive responses to abiotic stresses and classifies them accordingly into six categories: I) stabilization of cell walls and membranes; II) regulation of Na^+^ and K^+^ ratios; III) regulation of hormone levels in plants; IV) maintenance of photosynthesis; V) regulation of plant respiratory metabolism and improvement of root activities; and VI) induction of gene expressions and protein transcriptions for the stress resistance. Also, the progress and advances from the updated researches on exogenous calcium to alleviate seven abiotic stresses such as drought, flooding, salinity, high temperature, low temperature, heavy metals, and acid rain are outlined. Finally, the future research perspectives in agricultural production are discussed.

## Introduction

Plants are subject to a variety of biotic or abiotic adverse attacks and stresses throughout their entire growth, development and reproduction. These adversities can force plants to suffer from a mild osmotic, oxidative and/or ionic imbalance to serious biochemical and physiological disorders such as disruption of cell membranes, reduced enzyme activity, weakened photosynthesis and respiration, and decreased uptake of mineral elements (AbdElgawad et al., 2016; Zhang et al., 2022). To some degree, plants possess limited capabilities of improving their resilience, resistance or tolerance against some mild stresses through a series of morphological, physiological and molecular adjustments, but they are not strong or sufficient to survive from the severe ones. Therefore, Seeking for exogenous substances to induce or enhance such plant positive responses against irreversible adversities have become an ultimate goal for many researchers working on this field, and a promising, realistic and feasible approach in the current research attempts. So far, many exogenous substances have proven to be beneficial and effective in alleviation of plant abiotic stresses (Feng et al., 2023).

Among exogenous substances discovered to alleviate plant stresses, calcium ion (Ca^2+^) has proven to be more effective and the cost/benefit efficient, not only as an essential nutrient for the plant growth actively involving in a various metabolic activities and as an intracellular messenger for many signal transductions, such as abscisic acid (ABA), reactive oxygen species (ROS), nitric oxide (NO), et al. (Besson-Bard et al., 2008; Marcec et al., 2019; Shabbir et al., 2022). Different parts of a plant differ in their absorption, translocation and utilization of exogenous Ca^2+^. The exogenous Ca^2+^ absorbed by roots is mainly through a mass flow of soil water to the root surface and the apoplastids and coplastids in root vascular bundles for its upward movement due to plant transpiration (Zhou and Wang, 2007). Once being applied on foliage, exogenous Ca^2+^ enters mesophyll cells mainly through stomata, hydrophilic pores in the stratum corneum, and ectoplasmic filaments distributed on the leaf surface. Once inside plants, free Ca^2+^ moves through Ca^2+^ transport systems such as the sphinoplasmic Ca^2+^ outflow system (Ca^2+^-ATPase pump), Ca^2+^/H+ reverse transporter, and cytosolic Ca^2+^ influx system so that the Ca^2+^ concentration increases rapidly in tissues where Ca^2+^ is needed for normal metabolic functions or in response to environmental changes/stresses for an internal homeostasis in plants (Zhou and Wang, 2007). Ca^2+^ also serves as a cellular messenger when the Ca^2+^ concentration in cytoplasm is different from that in intercellular space available as a Ca^2+^ source. A high concentration of Ca^2+^ in a plant seemly acts as a transmitting signal for the activation of various metabolic and molecular activities. Calcium is inert, difficult to move around in plants, hardly to be reused by cells, and prone to bind to organic acids for a further transportation to and utilization in tissues and cells that really needs it. Moreover, any stresses such as drought, high temperature, low temperature and excessive rain affecting plant transpiration can seriously further reduce the calcium absorption and movement even if soil Ca^2+^ is available, in which case, a foliar application of exogenous calcium can be critically important for the plant stress tolerance. It has been reported that Ca^2+^ can help resist adverse irritations or damages to some extent in abiotically stressed plants through five mechanisms, including the regulation of sodium/potassium ion (Na^+^/K^+^) ratio and ABA concentration, stabilization of cell walls/plasma membranes, recognition of Ca^2+^/Ca^2+^-dependent protein kinases (CDPKs) system, and initiation of specific gene expression (Gong and Wang, 2011). However, the plants under stress are less capable of absorbing and translocating Ca^2+^, which could seriously impair plant tolerance and resistance against abiotic stresses. Therefore, an external application of different Ca^2+^ supplements has become a main stream of hot research frontlines to provide a sufficient quantity of usable Ca^2+^ for crops to fight against abiotic stresses (Heidari et al., 2019).

**Figure 1:**
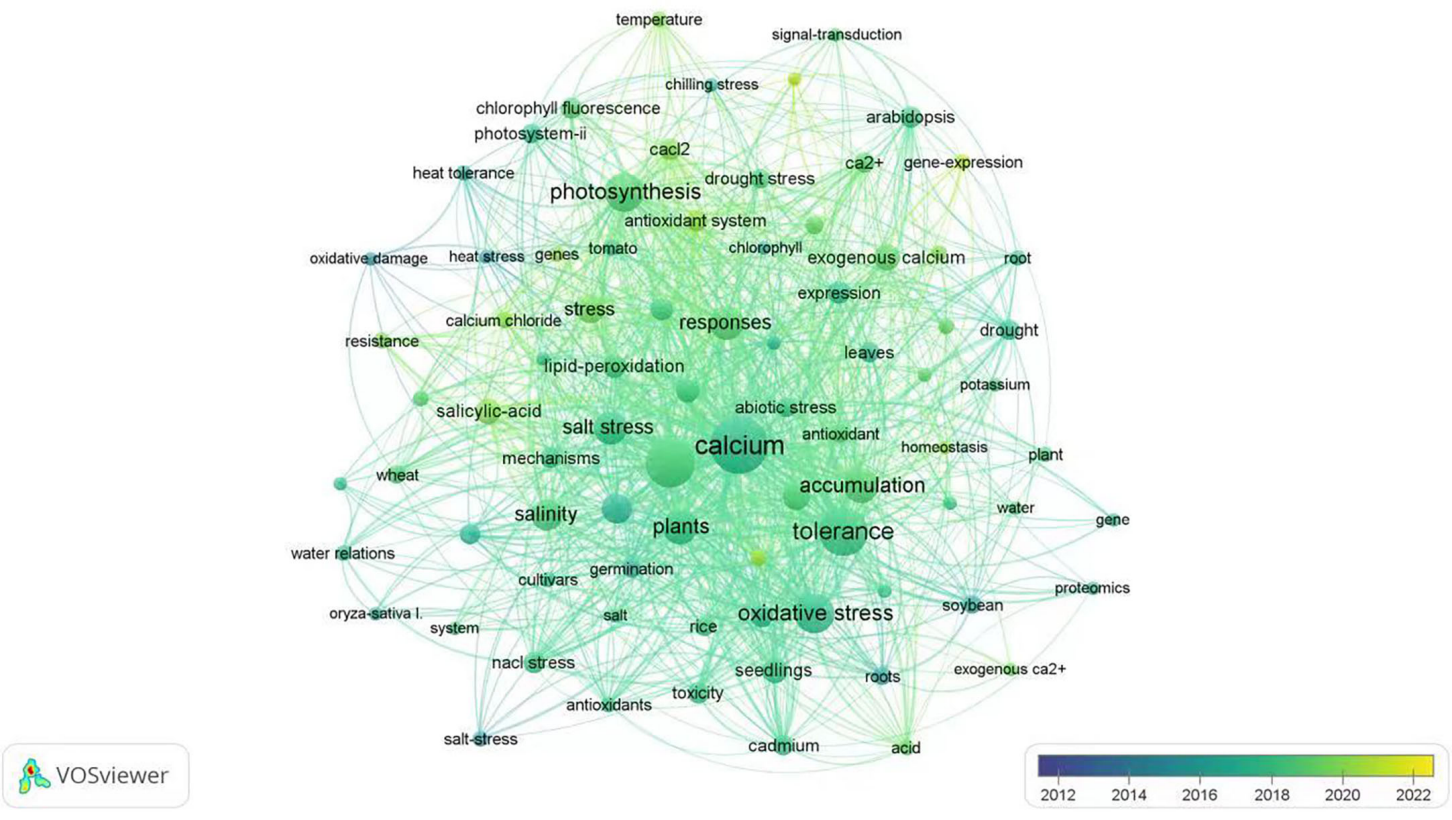
Hotspot analysis of English literature on “exogenous calcium” and “stress” in the last 10 years. The size of each dot represents the focal weight of each key word in the literature and the lines between two dots indicate their coupling relationships.

VOSviewer has been used to analyze all English literature published on the Web of science database for the last decade in the scientific research community. In this review, we also used this application to connect all important subjects (dots) under the keyword of “exogenous calcium” and “stress” to generate a hotspot map of international research reports (**Figure 1**). In the chart generated through VOSviewer, five research hotspots, such as “plant species”, “growth status”, “accumulation of substances”, “tolerance and response mode” are closely related to “exogenous calcium” and “stress”. As far as the application of exogenous calcium for alleviating plant stresses is concerned, most published papers are focused on the salt and drought stress while fewer studies have been conducted on the stress due to the high temperature, low temperature, flooding, heavy metal and acid rain. In terms of the mechanism pertaining to plant stress alleviation by utilization of exogenous calcium, “oxidative stress”, “photosynthesis”, “osmoregulation”, “gene and protein expression” and “membrane lipid peroxidation” have surfaced as research hotspots in recent years, among which “oxidative stress” and “photosynthesis” are the most studied areas in the past two years.

A number of advances have been made and many sophisticated instruments used in researches on application of exogenous calcium for plants to alleviate various stresses. Guo et al. (2021) used an inductively coupled plasma optical emission spectrometer to detect whether the exogenous calcium would increase K^+^ and Ca^2+^ abundance, decrease Na^+^ content in plants, and maintain the ion homeostasis in *Gleditsia sinensis* Lam. that was under salt stress through regulating the Na^+^/K^+^ ratio. He et al. (2015) applied the fluorescent and ultrastructural cytochemical method to determine if exogenous calcium had mitigated hazard effects of flooding stress on plant respiration by regulating the activity of respiratory metabolic enzymes in cucumber cells. Shi et al. (2014a) found that exogenous Ca^2+^ enhanced plant cold tolerance by promoting the differential expression of redox-related and cellular metabolism-pertaining proteins through a comparative proteomic and metabolomics analysis. Hu et al. (2018) indicated that an exogenous Ca^2+^ application not only had a positive effect on the integrity and function of plasma membrane but also alleviated peroxidative damages caused by draught stress on chloroplast and mitochondrial membranes. Exogenous Ca^2+^ have also proven to regulate the ABA content in plants under low temperature along with the hormone levels of gibberellic acid (GA), cytokinin (CTK), and indole-3-acetic acid (IAA) to maintain a balance (Liu et al., 2017). In addition, the degradation of plant chlorophylls, the damage to photosynthetic organs, and the stable performance of plant photosynthesis could be alleviated or prevented by exogenous Ca^2+^, which was demonstrated through using a portable chlorophyll fluorescence pulsometer (Li et al., 2022a). According to Gong and Wang (2011) the mode of actions with each exogenous calcium applied to alleviate plant stresses has much in common with the mechanism involving in plant responses to abiotic adversities, which is mainly through regulating ion balance, inducing expression of resistance genes and/or proteins, and increasing the enzyme activity and osmoregulatory substance content. However, the mechanisms involved in the plant stress alleviation are more complex and they are not easily or simply classified by one or two features based on regulating, modulating or participating in biological and physiological metabolisms or gene expression. For example, the mechanism associated with the recognition of Ca^2+^/CDPKs (Gong and Wang, 2011) mainly is through activating key enzymes and further inducing a series of complex physiological and biochemical responses such as production of more resistant proteins and enhancement of gene expressions for plant stress tolerance. So, the CDPKs recognition mechanism should be included into the category of the gene expression. Moreover, under flooding stress, exogenous calcium can also regulate the plant respiratory pathway to increase root activities for plants to withstand flooding stress (Yang et al., 2016; Han et al., 2019). Based on the relevant mechanisms of exogenous calcium on alleviating adversary stresses and the accumulative information shown in the hotspot analysis maps that is generated from the VOSviewer report for last 10 years, this paper intends to summarize the underlying mechanisms of exogenous Ca^2+^ involved in alleviation of plant abiotic stresses and tentatively place them into six major categories: I) stabilization of cell wall and membranes; II) regulation of Na^+^ and K^+^ ratios; III) regulation of hormone levels in plants; IV) maintenance of photosynthesis: V) regulation of plant respiratory metabolism and improvement of root activities; and VI) induction of gene expressions and protein transcriptions for the stress resistance. Since several outstanding papers have outlined other functional aspects of Ca^2+^ in plants such as its role as a signal messenger, its regulations as a molecular modulator in gene expressions, and its movement through transpiration, *etc*. (Edel and Kudla, 2016; Marcec et al., 2019; Wang X. et al., 2019; Liu H. et al., 2022), this review has ultimately summarized in detail the research progresses made on studies of exogenous Ca^2+^ and its effect on alleviation of seven abiotic stresses, drought, flooding, salt, high temperature, low temperature, heavy metals, and acid rain. At the end, a discussion of perspectives for developing experimental approaches that would take those mechanisms into account for use of different exogenous Ca^2+^ in increasing plant tolerance against abiotic stresses is provided.

## Advances in application of exogenous Ca^2+^ under abiotic stresses

### Drought stress

Recent studies on the drought alleviation by exogenous Ca^2+^ were conducted on 15 plant species and outlined in detail (**Table 1**), concluding that it actively involved in keeping water content and balance under check through the mechanism I, III, IV and VI when water deficit is encountered in plants under drought stress. Drought stress is one of the most important adversities causing crop yield reduction worldwide (Iqbal et al., 2020), causing imbalance in water metabolism in plants, affecting photosynthesis and bringing adverse effects on plant growth and development. Qin et al. (2019) found that exogenous Ca^2+^ help promote plants under drought stress to accumulate endogenous Ca^2+^, especially free Ca^2+^ content in datura plants that bind the calmodulin and thus directly or indirectly regulate intracellular related enzyme activities and cellular functions to improve seed germination. Hu et al. (2018) found that exogenous Ca^2+^ treatment under drought stress can stabilize the structure and function of chloroplast, mitochondrial and endosomal systems in chloroplasts, maintain net photosynthetic rate and gas exchange, alleviate the degradation of photosynthate, and ensure the normal operation of PSII. Li et al. (2012) found that exogenous Ca^2+^ could reduce the stomatal aperture of honeysuckle for adaptation to drought conditions. In addition, Naeem et al. (2017) indicated that the exogenous Ca^2+^ treatment could increase the relative water content in maize and compensate a water deficit caused by drought to some extent.

**Table 1:** List of optimal concentrations of exogenous calcium used to alleviate drought stress on different plants.

Type of Calcium	Method of application	Optimum concentration	Test crops	Plant responses against drought stress	Reference
CaCl_2_	Foliar spraying	10 mM	*Zoysia japonica* Steud.	Increase of Chl content, net photosynthetic rate (Pn), chlorophyll fluorescence, antioxidant enzyme activities, lipid peroxidation, and proline (Pro) content.	Xu et al. (2013)
Calcium Nanoparticles	Foliar spraying	100 mg L^-1^	*Brassica napus* L.	Increase of non-photochemical quenching (NPQ), antioxidative defense enzymes (SOD, POD, CAT, APX), non-enzymatic components (protease, lipoxygenase, Pro, total soluble proteins, endogenous hormonal biosynthesis), and secondary metabolites.	Ayyaz et al. (2022)
CaCl_2_	Irrigation	100 mg kg^-1^	Mongolian pine (*Pinus sylvestris* var. Mongolica)	Affecting the growth, photosynthetic characteristics and antioxidant enzyme activities, increasing the net photosynthetic rate, transpiration rate (Tr), stomatal conductance, chlorophyll content, chlorophyll fluorescence parameters, soluble sugar, starch and antioxidant enzyme activities.	Li et al. (2022b)
CaCl_2_·2H_2_O	Foliar spraying	40 mg L^-1^	Maize (*Zea mays* L.)	Causing a noticeable increase in the activity of SOD, CAT and APX, considerably reduced MDA accumulation.	Naeem et al. (2017)
CaCl_2_	Watering	10 mM	Wheat (*Triticum aestivum* L.)	Promoting seed germination, shoot growth, and chlorophyll content, enhancing higher photosynthetic capacity and reducing electrolyte leakage, MDA content as well as H_2_O_2_ accumulation.	Bhardwaj et al. (2018)
CaCl_2_	Irrigation	30 mM	Honeysuckle (*Lonicera japonica* Thunb.)	Increase of the content of chlorophyll, soluble sugar, Pro, catalase enzyme activity and the photosynthetic relating enzymes.	Li et al. (2012)
CaCl_2_	Foliar spraying	100 μM	*Camellia sinensis* (L.) O. Ktze.	Increase in dry weight, Pro and phenolic content of leaves with a decrease in H_2_O_2_ and lipid peroxidation along with an increase of activities of enzymes such as SOD, CAT, pyruvate oxidase (POX) and GR, for improvement in drought recovery potential.	Upadhyaya et al. (2011)
CaCl_2_	Foliar spraying	10 mM	Tobacco (*Nicotiana tabacum* L.)	Stabilizing the structure and function of the chloroplasts, mitochondria, and endomembrane system in the mesophyll cells, maintaining normal leaf net photosynthetic rate and gas exchange, alleviating the degree of photosynthetic pigment degradation, and increasing the electron transfer energy in the leaves in PSII.	Hu et al. (2018)
CaCl_2_	Wetting filter paper	20 mM	Datura (*Datura stramonium* L.)	Regulating the activity of relevant enzymes and cell function.	Qin et al. (2019)
CaCl_2_	Hydroponics	20 mM	*Cyclobalanopsis glauca* (Thunberg) Oersted	Increasing the relative water content (RWC) and water potential (WP), reducing the H_2_O_2_ and MDA content, alleviating drought-induced oxidative activities of antioxidant enzymes, and enhancing the accumulation of osmoregulation substances, Pn, stomatal conductance (Gs), Tr, and chlorophyll content.	Xue et al. (2018)
CaCl_2_	Hydroponics	11 mM	Maize (*Zea mays* L.)	Mitigating the inhibition of aquaporin expression and/or activity levels *via* osmotic pathway.	Wu et al. (2012)
CaCl_2_	Foliar spraying	15 mM	*Brassica napus* L.	Increasing chlorophyll content.	Khan et al. (2017)
CaSO_4_	Foliar spraying	1%	Tomato (*Solanum lycopersicum* L.)	Increasing the magnesium and chlorophyll and carbohydrate level.	Birgin et al. (2021)
CaCl_2_·2H_2_O	Foliar spraying	50 mg L^-1^	Maize (*Zea mays* L.	Increasing protein, ash, carbohydrates, starch, total sugars, and ionic contents and K^+^ amount.	Abbas et al. (2021)
Calcium Lactate	Foliar spraying	1.5 g L^-1^	Lettuce (*Lactuca sativa* L.)	Increasing the content of anthocyanin, total phenols, flavonoids, N, promoting CAT and POX enzymes activity, and increasing the dry matter production as well as the water use efficiency.	Khani et al. (2020)
CaCl_2_	Foliar spraying	10 mM	Sweet pepper (*Capsicum annuum* L.)	Increasing total soluble sugars and soluble protein and reducing the Pro concentration.	Manaf et al. (2017)
CaCl_2_	Foliar spraying	5 mM	Peony (*Paeonia* section *Moutan* DC.)	Inducing the enzyme activity of the antioxidant enzyme system, and reducing the accumulation of reactive oxygen species (ROS), REC and Pro content, and minimizing the degree of cell membrane damage.	Zhang et al. (2019b)
CaCl_2_	Foliar spraying	10 mM	Sesame (*Sesamum indicum* L.)	Increasing the chlorophyll, potassium and phosphorus content.	Heidari et al. (2019)
CaCl_2_	Soaking	50 mM	Barley (*Hordeum vulgare* L.)	Increasing Mg^2+^, Zn^2+^, Ca^2+^ levels, RWC and gas exchange parameters, and promoting the Gs, chlorophyll and carotenoid content.	Kaczmarek et al. (2017)

### Flooding stress

Recent research findings have demonstrated that exogenous Ca^2+^ (CaCl_2_ only) is partially or completely participated in all structural, physiological, biochemical and genetic adjustments against flooding stress that causes temporary or prolonged hypoxia in crop roots due to lack of oxygen. The impairments or damages of hypoxia mainly include a blockage of the mitochondrial electron transport chain, a reduced aerobic respiration and an enhanced anaerobic respiration. Thus, a large amount of toxic ethanol, acetic acid, pyruvic acid and other substances are subsequently produced and accumulated in plants. Recent studies have proven that the exogenous Ca^2+^ treatment could have: 1) effectively minimized the ethanol, acetic acid and pyruvate content to mitigate their toxic effects in peony plants under flooding stress through reducing activities of the lactate dehydrogenase (LDH) and pyruvate decarboxylase (PDC), and increasing the alcohol dehydrogenase (ADH), malate dehydrogenase (MDH), and glucose-6-phosphate dehydrogenase (G-6-PDH) (Fan, 2019); 2) improved the catalytic capacity of pepper pentose phosphate so to produce more adenosine-triphosphate (ATP) and nicotinamide adenine dinucleotide (NADPH) and to improve plant respiratory metabolism (Yang et al., 2016); 3) promoted glycolysis and the accumulation of enzymes in the tricarboxylic acid cycle by reducing the peroxidation level of cucumber seedlings and enhanced the activity of mitochondrial antioxidant enzymes to promote the metabolism of cucumber roots and the transport of Ca^2+^ and K^+^ plasma, thus improving the hypoxia tolerance of cucumber (He et al., 2015); and 4) reduced polyamine degradation in muskmelon seedlings under anoxic conditions by promoting nitrate uptake and accelerating its conversion to amino acids, heat-stable proteins, or polyamines (Gao et al., 2011). Relevant studies of exogenous Ca^2+^ in mitigation of plant flooding stress are conducted on five crop species (**Table 2**).

**Table 2:** List of optimal concentrations of exogenous calcium used to alleviate flooding stress on different plants.

Type of Calcium	Method of application	Optimum concentration	Test crops	Plant responses against flooding stress	Reference
CaCl2	Foliar spraying	0.3 mM	Peony (*Paeonia suffruticosa* Andr.)	Slowing down the decrease of chlorophyll content, the increase of conductivity and MDA, and enhancing the activity of protective enzymes (SOD, CAT, APX)	Pan et al. (2018)
CaCl2	Foliar spraying	0.3 mM	Peony (*Paeonia suffruticosa* CV. ‘Luyanghong’	Reducing the activities of lactate dehydrogenase (LDH), pyruvate decarboxylase (PDC), and the content of ethanol, acetaldehyde and pyruvate, and increasing ethanol dehydrogenase (ADH), malic dehydrogenase (MDH), glucose-6-phosphate dehydrogenase (G-6-PDH) activities.	Fan (2019)
CaCl2	Foliar spraying	10 mM	Pepper (*Capsicum annum* L.)	Increasing the chlorophyll content, CAT, POD, SOD, GR, ADH, MDH activity and total soluble sugar, and reducing the relative conductively ratio, LDH activity, OH and MDA content.	Liu et al. (2015b)
CaCl2	Hydroponics	4 mM	Cucumber (*Cucumis sativus* L.)	Reducing the level of ROS, increasing the activity of antioxidant enzymes in mitochondria under hypoxia, and enhancing the accumulation of enzymes involved in glycolysis and the tricarboxylic acid (TCA) cycle.	He et al. (2015)
CaCl2	Hydroponics	6 mM	Muskmelon (*Cucumis melo* L. var. reticulates Naud.)	Accelerating its transformation into amino acids, heat-stable proteins or polyamines, as well as by decreasing polyamine degradation.	Gao et al. (2011)
CaCl2	Foliar spraying	10 mM	Pepper (*Capsicum annuum* L.)	Regulating the content of osmotic substances, antioxidant activity, root respiration, and metabolism.	Yang et al. (2016)
CaCl2	Hydroponics	4 mM	Cucumber (*Cucumis sativus* L.)	Enhancing electron transport capacity and reducing hypoxic damages.	He et al. (2018)
CaCl2	Hydroponics	4 mM	Cucumber (*Cucumis sativus* L.	Enhancing the gene expression of enzymes involved in glycolysis, the TCA cycle, fermentative metabolism, nitrogen metabolism, and ROS.	He et al. (2012)
CaCl2	Foliar spraying	10 mM	Pepper (*Capsicum annuum* L.	Maintaining the intactness of the cell wall in roots, cell membrane, and pulp cavity, reducing cell injury and stimulated the expression level of ACO-1, ADH-1, CAT-2, and PK genes.	Ou et al. (2017)
CaCl2	Hydroponics	50 mM	Soybean (*Glycine max* L.)	Protecting cell walls, hormone metabolisms, protein synthesis, and DNA synthesis from impairments in soybean roots.	Oh et al. (2014)

### Salt stress

Up to date, 19 plant species have been evaluated for their tolerance against different salt stresses through using various forms of exogenous Ca^2+^ in different application methods such as watering, hydroponic, soaking and foliar spray. The outcome from those studies showed that their overall mechanisms are diversified and complex mainly to maintain the ion balance and avoid the plant osmosis from impairment. Salt stress causes an ionic imbalance in plants due to the accumulation of Na^+^ and a great loss of Ca^2+^ and K^+^. It also causes osmotic impairments resulting in an oxidative disturbance by an excessive accumulation of ROS that affected the photosynthesis-related activities of electron transportation, phosphorylation, and dark reaction-involved enzymes (Shu et al., 2012; Manaa et al., 2014). Guo et al. (2021) found that exogenous Ca^2+^ promoted the function of K^+^ channels and its uptake through the root plasma membrane, reduced the permeability of the plasma membrane for Na^+^ pumping so to decrease the accumulation of passive Na^+^ inward flow. Li et al. (2022a) indicated that the exogenous Ca^2+^ treatment could increase the maximum photochemical efficiency (Fv/Fm) under salt stress in Mongolian pines and 10 mM exogenous Ca^2+^ could promote the growth of *Salix matsudana* Koidz seedlings and increase their stomatal conductance, transpiration rate (Tr) and net photosynthetic rate. Li et al. (2022c) also showed that exogenous Ca^2+^ significantly up-regulated genes encoding phospholipase C, inositol-3-phosphate synthase, and phosphatidylserine decarboxylase to stabilize cell membranes, up-regulated the expression of PsbQ, PsbP, and Psb28 subunits on encoded PSII, and protected PSII to increase photosynthetic rate of *Pennisetum Giganteum*, revealing the connections between the gene regulation and biochemical metablisms. Zehra et al. (2012) found that the concentration of exogenous Ca^2+^ required for alleviation of salt stress in *Phragmites karka* seeds varied. Relevant studies on exogenous Ca^2+^ mitigation of salt stress in plants involved 19 plant species, as detailed in **Table 3**.

**Table 3:** List of optimal concentrations of exogenous calcium used to alleviate salinity stress in different plants.

Type of Calcium	Method of application	Optimum concentration	Test crops	Plant responses against salinity stress	Reference
CaCl_2_	Watering	10 mM	*Pennisetum Giganteum*	Up-regulating differential genes significantly enriched in carbohydrate metabolism, photosynthesis, lipid metabolism, thylakoid, *etc*., from the GO enrichment profiling and up-regulating genes for photosynthesis antenna proteins, photosynthesis and other metabolic processes from the KEGG enrichment profile.	Li et al. (2022c)
CaCl_2_	Hydroponics	10 mM	*Gleditsia sinensis* Lam.	Attenuating the cytotoxicity caused by Na^+^ under salt stress and promoting the equilibrium of ion homeostasis.	Guo et al. (2021)
Prohexadione-calcium	Foliar spraying	100 mg L^-1^	Soybean (*Glycine max* L.)	Regulating plant phenotype, photosynthetic apparatus, antioxidant defense, and osmoregulation.	Feng et al. (2021)
Ca_2_SiO_4_	Foliar spraying	1 mM	Okra (*Abelmoschus esculentus* L.)	Reducing the Na^+^ concentration in the leaf apoplast.	Qadir et al. (2017)
CaCl_2_	Soaking	6 mM	Soybean (*Glycine max* L.)	Enriching signal transduction, energy pathway and transportation, promoting protein biosynthesis, inhibiting proteolysis, redistributing storage proteins, regulating protein processing in endoplasmic reticulum, enriching antioxidant enzymes and activating their activities, accumulating secondary metabolites and osmolytes.	Yin et al. (2015)
CaCl_2_	Soaking	2 mM	Choysum (*Brassica* *rapa* var. *parachinensis*)	Enhancing hormonal regulation by decreasing the abscisic acid (ABA) levels with a concomitant increase of GAs (especially GA4) levels and promoting early germination, decreasing Na^+^ and increasing K^+^ contents so to maintain a balanced Na^+^/K^+^ ratio.	Kamran et al. (2021)
CaCl_2_	Foliar spraying	10 mM	*Thymus vulgaris* L.	Enhancing the activity of the antioxidant enzymes of the ascorbate glutathione cycle to allow a better protection of the cell membranes from reactive oxygen species.	Zrig et al. (2021)
CaCl_2_	Soaking	5 mM	Maize (*Zea mays* L.)	Upregulating the expression of all key carotenogenic genes.	He et al. (2020)
CaCl_2_	Hydroponics	2 mM	Rice (*Oryza sativa* L.)	Improving ROS and methylglyoxal detoxification by improvement of the antioxidant defense and glyoxalase systems.	Rahman et al. (2016)
CaCl_2_	Soaking	5 mM	*Sorghum bicolor* (L.) Moench	Counteracting oxidative stress and improving Na^+^/K^+^ ratio.	Mulaudzi et al. (2020)
CaCl_2_	Soaking	10 mM	*Festuca ovina* L.	Improving germination, reducing Na^+^ binding to cell walls, and alleviating membrane leakages.	Salahshoor and Kazemi (2016)
CaCl_2_	Soaking	10 mM	*Phragmites karka* (Retz.) Trin, ex. Steud.	Modulating seed germination responses, maintains Na^+^ and K^+^ homeostasis *via* SOS (salt overly sensitive) pathway, increasing the activity of antioxidant enzymes.	Zehra et al. (2012)
CaCl_2_	Soaking	10 mM	Tomato (*Solanum lycopersicum* L.)	Improving the seedling growth, RWC and stabilizing membrane stability.	Tanveer et al. (2020)
CaCl_2_	Watering	15 mM	*Nitraria sibirica* Pall.	Adjusting hormone balance through increasing ABA, IAA and gibberellic acid (GA) contents.	Wu et al. (2022)
CaCl_2_	Soaking	10 mM	Rice (*Oryza sativa* L.	Elevating levels of catalase and ascorbate peroxidase activity, increasing RWC, improving chl-a, chl-b and total chls conten.	Roy et al. (2019)
CaCl_2_	Watering	10 mM	Foxtail millet (*Setaria italica* L.)	Upregulating the expression of APX, SOD and CAT.	Han et al. (2019)
CaSO_4_	Watering	10 mM	Tomato (*Solanum lycopersicum* L.)	Improving chlorophyll content and maintaining other morphological features and physiological metabolisms.	Khursheda et al. (2015)
Ca	Hydroponics	10 mM	Mongolian pine (*Salix matsudana* Koidz.)	Increasing the photosynthetic parameters, photosynthetic pigment content and photosynthetic product synthesis.	Li et al. (2022a)
Ca(NO_3_)_2_	Watering	2 mM	Soybean (*Glycine max* L.)	Maintaining osmoregulation and antioxidant metabolism.	Elkelish et al. (2019)
CaCl_2_	Hydroponics	10 mM	Cucumber (*Cucumis sativus* L.)	Increasing free Pro, SOD activity, relative growth rate of plant height, and stem volume, and K^+^/Na^+^ and K^+^/Ca^2+^, and decreasing MDA content.	Wang et al. (2021)
CaCl_2_	Hydroponics	10 mM	Sour jujube (*Ziziphus jujuba* Mill. var. *spinosa* (Bunge) Hu ex H. F. Chow)	Educing Na^+^ concentrations and increasing K^+^, Ca^2+^, and Mg^2+^ concentrations.	Jin et al. (2017)
Ca(NO_3_)_2_	Hydroponics	17.5 mM	Wheat (*Triticum aestivum* L.)	Facilitating the maintenance of ion homeostasis.	Tian et al. (2015)
CaCl_2_	Hydroponics	10 mM	Hyacinth bean (*Lablab purpureus* (L.) Sweet)	Enhancing levels of H_2_O_2_, MDA, glutathione (GSH), ASC, TSS and photosynthetic pigments and increasing the activity of metabolic enzyme β-Amylase.	D’souza and Devaraj (2013)
CaSO_4_	Watering	40 mM	Tomato (*Lycopersicon esculentum* Mill.)	Maintaining of high K^+^/Na^+^ ratio in leaves.	Henry et al. (2021)

### High temperature stress

While being applied to plants, exogenous Ca^2+^ can prevent or mitigate a possible light damage caused by high temperature that assumingly disrupts the photosynthetic function of plants and severely affects their photosynthesis efficiency. Wang et al. (2022) found that the exogenous Ca^2+^ treatment significantly increased the chlorophyll (Chl) content, net photosynthetic rate (An), Tr, stomatal conductance (Gs), and antioxidant enzyme activities such as SOD, POD, ascorbate peroxidase (APX), and proline (Pro), as well as the content of osmoregulatory substances such as soluble sugars and soluble proteins to improve the heat tolerance of rosebay. Sun et al. (2015) indicated that exogenous Ca^2+^ could increase the ribulose-1,5-bisphosphate carboxylase (Rubisco) activity and leaf Fv/Fm of *Capsicum fructescens* L. to alleviate the photoinhibition to enhance the stomatal conductance and carbon assimilation efficiency. Tiwari et al. (2016) demonstrated that the exogenous Ca^2+^ treatment upregulated the levels of heat shock genes groEL and groES to maintain cell viability under high temperature stress. Bhatia and Asthir (2014) concluded that the exogenous Ca^2+^ treatment maintained the growth of wheat seedlings under high temperature stress by altering the carbohydrate metabolism in wheat seeds and increasing the total sugars through reducing sugar-metabolism-related a-amylase and β-amylase activities. In addition, Naeem et al. (2020) showed that exogenous Ca^2+^ was also involved in the process of adjusting leaf surface structure and configuration to dissipate heat by regulating the conductance of plant stomata for the purpose of alleviating heat stress in plants. Relevant studies on exogenous Ca^2+^ mitigation of heat stress in plants involved 11 plant species, as detailed in **Table 4**.

**Table 4:** List of optimal concentrations of exogenous calcium used to alleviate heat stress in different plants.

Type of Calcium	Method of application	Optimum concentration	Test crops	Plant responses against heat stress	Reference
CaCl_2_	Foliar spraying	10 mM	*Capsicum frutescens* L.	Improving the activity of ROS scavenging enzymes and the contents of some osmoregulation substances.	Sun et al. (2015)
CaCl_2_	Foliar spraying	20 mM	*Phalaenopsis aphrodite* H. G. Reichenbach	Increasing the SOD, POD and CAT activities, and the content of Pro, soluble sugar, soluble protein, total chlorophyll and carotenoids contents and decreasing MDA content.	Yang and Yang (2021)
CaCl_2_	Foliar spraying	5 mM	Common bean (*Glycine max* L.)	Up-regulating the enzymatic activities, and down-regulating the MDA accumulation and electrolyte leakage in plant leaf tissues, enhancing the accumulation of sugars (glucose, fructose, inositol, and raffinose)	Naeem et al. (2020)
CaCl_2_	Soaking	5 mM	Wheat (*Triticum aestivum* L.)	Alleviating the inhibition of sucrose and starch metabolism.	Bhatia and Asthir (2014)
CaCl_2_	Foliar spraying	20 mM	Tobacco (*Nicotiana tabacum* L.)	Improving stomatal conductance and the thermostability of oxygen-evolving complex (OEC).	Tan et al. (2011)
Ca(NO_3_)_2_	Hydroponics	6 mM	Peanut (*Arachis hypogaea* L.)	Protecting the photosynthetic system by accelerating the repair of D1 protein and improving the de-epoxidation ratio of the xanthophyll cycle.	Yang et al. (2013)
CaCl_2_	Foliar spraying	100 μM	*Rosa rugosa* Thunb.	Regulating photosynthesis, the antioxidant system, and osmotic substances.	Wang et al. (2022)
CaCl_2_	Foliar spraying	20 mM	*Camellia sinensis* (L.) O. Ktze.	Up-regulating 299 and down-regulating 624 of 923 differentially expressed genes (*DEGs*) relating to signal transduction, transcription, and post-translation, respectively.	Wang M et al. (2019)
CaCl_2_	Mixing into the medium	0.25 mM	Cyanobacterium *Anabaena* PCC 7120	Activating heat shock genes (*groEL* and *groES*) in Ca^2+^-supplemented cultures.	Tiwari et al. (2016)
Ca(NO_3_)_2_	Foliar spraying	4 mM	Spinach (*Spinacia oleracea* L.)	Increasing antioxidant enzyme activities, soluble sugar levels, SOD, CAT and POD, and reducing membrane leakages.	uz Zaman et al. (2022)
CaCl_2_	Foliar spraying	10 mM	Wheat (*Triticum aestivum* L.)	Reducing lipid peroxidation and increasing the total antioxidant capacity of the cell system.	Goswami et al. (2015)

### Low temperature stress

Low temperature as one of the main abiotic stresses reduces the cell membrane fluidity and enzyme activities before the temperature reaches a freezing point, inhibits plant physiological metabolic activities, and affects seed germination and seedling growth through the mechanism I, III, IV, VI as described in **Table 5**. The exogenous Ca^2+^ treatment could help leaves adjust their structure and configuration, promote the operation of cyclic electron transport and enhance the lutein cyclic de-cyclic oxidation in cells to alleviate photoinhibition in tomato plants (Zhang et al., 2014). It also mitigated the damage to chloroplasts due to the low temperature and promote the export of nonstructural carbohydrates to maintain normal plant photosynthesis in peanut seedlings (Wu et al., 2020b). Zhang et al. (2020) indicated that an exogenous application of CaCl_2_ significantly increased chlorophyll fluorescence indicators (Fv/Fo, Fv/Fm) and the photosynthetic rate in maize, while Liu J et al. (2022) demonstrated that the same treatment onto onion plants reduced cell wall porosity and lowered intracellular ice nucleus temperature. Shi et al. (2014a) concluded that adding exogenous Ca^2+^ enhanced ROS scavenging through increasing the activity of antioxidant enzymes and non-enzymatic GSH to maintain intracellular ROS at a low level. Relevant studies on exogenous Ca^2+^ in mitigation of low temperature stress in plants have been reported on nine plant species (**Table 5**).

**Table 5:** List of optimal concentrations of exogenous calcium used to alleviate low temperature stress in different plants.

Type of Calcium	Method of application	Optimum concentration	Test crops	Plant responses against low temperature stress	Reference
CaCl_2_	Foliar spraying	10 mM	*Eucalyptus urophylla ×E. grandis*	Reducing the MDA content but increasing the ABA level, the content of Pro, soluble sugar, cytokinin (CTK), and GA and the activities of CAT, POD, SOD.	Liu et al. (2017)
CaCl_2_	Foliar spraying	15 mM	*Elymus nutans* Griseb.	Inducing the expression levels of nine antioxidant enzyme genes.	La-mu et al. (2021)
CaCl_2_	Foliar spraying	27 mM	Tomato (*Solanum lycopersicum* L.)	Increasing the Pn, effective quantum yield of PSII [Y(II)], and photochemical quenching (qP), improving carbon fixation capacity, plastoquinone pools, linear and cyclic electron transports, xanthophyll cycles, and ATPase activity.	Zhang et al. (2014)
CaCl_2_	Watering	25 mM	Spinach (*Spinacia oleracea* L.)	Reducing K^+^, Mg^2+^, and total ion leakage, alleviating oxidative stress, and enhancing PSII potential quantum yield/energy trapping efficiency (Fv/Fm).	Min et al. (2021)
CaCl_2_	Watering	50 mM	Onion (*Allium fistulosum* L.)	Accumulating galacturonic acid, stabilizing the cell wall, and preventing cell membrane leakages.	Liu J et al. (2022)
CaCl_2_	Foliar spraying	15 mM	Peanut (*Arachis hypogaea* L.)	Protecting the photosystems from photoinhibition by facilitating cyclic electron flow (CEF) and decreasing the proton gradient (ΔpH) across thylakoid membranes.	Wu et al. (2020b)
CaCl_2_	Foliar spraying	27 mM	Tomato (*Solanum lycopersicum* L.)	Improving photosynthesis.	Liu et al. (2015a)
CaCl_2_	Foliar spraying	15 mM	*Peanut (Arachis hypogaea* L.)	Restoring temperature-dependent photosynthesis feedback inhibition due to improved growth/sink demand.	Song et al. (2020)
CaCl_2_	Foliar spraying	15 mM	*Peanut (Arachis hypogaea* L.)	Improving the stomatal conductivity and mitigating the decline of photosynthetic rate.	Liu et al. (2013)
CaCl_2_	Foliar spraying	7 mM	*Piper nigrum* L.	Improving the activity of antioxidant enzymes, increasing the soluble sugar content and reducing the MDA content.	Wu et al. (2020a)
CaCl_2_	Soaking	80 mM	*Maize (Zea mays* L.)	Protecting the function and structure of the membrane and photosystems, improving antioxidant enzyme activity and increasing osmotic regulatory substances.	Zhang et al. (2020)
CaCl_2_	Mixing into the medium	5 mM	Bermuda grass (*Cynodon dactylon* L. Pers.)	Alleviating the ROS burst and cell damage.	Shi et al. (2014a)

### Heavy metal stress

All researches on exogenous Ca^2+^ in alleviation of heavy metal stress in plants have indicated that additional Ca^2+^ limits the uptake, movement and distribution of excessive heavy metals that might accumulate to a toxic level through five mechanisms except the mechanism V summarized in **Table 6**. Most studies have been focused on alleviation of Cd toxicity by an exogenous Ca^2+^ application. López-Climent et al. (2014) determined that exogenous Ca^2+^ had attenuated the Cd uptake in citrus through enhancing the metabolism to detoxicate harmful ions, which promoted the GSH synthesis, and thus increased endogenous GSH levels of the phytochelatin (PC) biosynthesis for the Cd detoxication. According to Shi et al. (2014b), exogenous Ca^2+^ increased the mitotic index and decreased the chromosomal aberration rate of *Wedelia trilobata* L. to transport Cd out of stressed cells. Li et al. (2021) suggested that exogenous Ca(OH)_2_ was more effective than CaCl_2_ in increasing the quantity of the Ca^2+^ channel protein (CC), ATPase, cationic/H^+^ antiporter (CAXs) and membrane transporter protein in *Panax notoginseng* plants under Cd stress. A proper application of Ca(OH)_2_ was also reported to increase soil pH, decrease the toxicity of heavy metals, and reduced the uptake of Cd by plants (Zu et al., 2020). Issam et al. (2012) pretreated the Faba bean (*Vicia faba* L.) foliage with exogenous Ca^2+^ and proved that the membrane integrity and lipid/fatty acid distribution were protectively stabilized to tolerate heavy metal stress. Jiang et al. (2022) hypothesized that exogenous Ca^2+^ might have reduced the toxicity of Pb through depositing Pb^2+^ in the cell wall, which might had nothing to do with soil properties. Relevant studies on exogenous Ca^2+^ mitigation of heavy metal stress in plants involved 11 plant species, as detailed in **Table 6**.

**Table 6:** List of optimal concentrations of exogenous calcium used to alleviate heavy metal stress in different plants.

Type of Calcium	Type of heavy metal	Method of application	Optimum concentration	Test crops	Plant responses against heavy metal stress	Reference
Ca(OH)_2_	Cd	Mixing into the soil	360 mg kg^-1^	*Panax notoginseng* (Burk.)	Promoting activities of Ca^2+^ channel protein (CC), ATPase and cationic/H^+^ antiporter.	Li et al. (2021)
Ca(NO_3_)_2_	Cd	Hydroponics	2 mM	*Catharanthus roseus* (L.) G. Don	Alleviating Cd-induced toxicity, including browning and rot roots, oxidative stress and internal Cd(II) accumulation.	Chen et al. (2018)
CaCl_2_	Cd	Soaking	2%	Faba bean (*Vicia faba* L.)	Improving soluble protein, total membrane lipid contents, fatty acid composition and the activities of SOD, CAT, GPX, lipoxygenase, decreasing the contents of Cd and MDA.	Issam et al. (2012)
Ca(NO_3_)_2_	Cd	Mixing into the medium	10 mM	Lettuce (*Lactuca sativa* var. *ramosa* Hort.)	Competing with Cd for binding and absorption sites in roots.	Zorrig et al. (2012)
CaO	Cd	Watering	7.5 mM	Citrus (*Poncirus trifoliata* L. Raf × *Citrus sinensis* L. Osb)	Reducing Cd^2+^ uptake into roots and also increasing GSH content.	López-Climent et al. (2014)
CaCl_2_	Cd	Mixing into the medium	30 mM	*Wedelia trilobata* L.	Enhancing the mitotic index and reducing the rate of chromosomal aberration in root tip cells.	Shi et al. (2014b)
CaCl_2_	Cd	Mixing into the soil	180 mg kg^-1^	*Panax notoginseng* (Burk.)	Increasing the biomass, saponin and flavonoid yields.	Zu et al. (2020)
Ca(OH)_2_	Cd	Mixing into the soil	1125 kg hm^-2^	*Panax notoginseng* (Burk.)	Increasing the biomass, saponin and flavonoid yields.	Zu et al. (2020)
CaCl_2_	Cd	Mixing into nutrient solution	50 mM	*Brassica juncea* L.	Enhancing the concentration of essential elements and decreasing Cd accumulation.	Ahmad et al. (2015)
CaCl_2_	Pb	Watering	400 mg L^-1^	Water spinach (*Ipomoea aquatica* Forssk.)	Increasing chlorophyll content, decreasing MDA content, promoting soil cation exchange capacity and the activity of soil Urease and CAT.	Jiang et al. (2022)
Ca(NO_3_)_2_	Cd, Mn	Mixing into the medium	4.4 mM	Lichen (*Scenedesmus quadricauda*)	Increasing antioxidative enzyme activities (SOD, APX, and CAT) and depleting ROS contents.	Kováčik and Dresler (2018)
CaCl_2_	Cd	Hydroponics	5 mM	*Arabidopsis thaliana* (L.) Heynh.	Promoting the upward translocation of Cd and changeing its distribution in leaves.	Zeng et al. (2017)

### Acid rain stress

Exogenous Ca^2+^ has been tried and studied on for its possible application when plants are under an acid rain situation. So far, Ca^2+^ has proven to be effective through the mechanism I, III, IV and VI. Acid rain is defined as any precipitation with a pH less than 5.6 due to a large amount of acid substances accumulated in the atmosphere mainly through human activities. Damages caused by acid rain on plant leaves directly breakdown the protective surface of leaves, destroy the integrity of inner plant cell membranes, and cause the organelle dysfunction. A persisted and long duration of acid rain can lead to a serious and catastrophic impairments on plant structural compositions (Zhang et al., 2021), but there have been limited studies working on use of the exogenous Ca^2+^ to improve plant cell membranes. One of them indicated that exogenous Ca^2+^ increased the H - ATPase activity with the soybean plasma membrane, kept the membranes unharmed, and initiated the GmPHA1 gene expression to generate more nutrient uptakes such as N, P, K, and Mg to keep chlorophylls from degradation (Liang and Zhang, 2018). Exogenous Ca^2+^ was also found to change the quantity of different forms of calcium such as water-soluble organic calcium, calcium pectinate, and calcium phosphate in *Brassica napus* against the acid rain stress (Cong, 2018). Also, while being evaluated on six forest tree species, exogenous Ca^2+^ was able to reduce the negative effects of acid rain stress imposed on the seed germination, seedling growth, leaf chlorophyll content, and plant photosynthesis (Liu et al., 2011).

### Some perspectives on future research

As we have discussed above, draught, flooding, salt, high and low temperature, heavy metals and acid rain are seven commonly encountered abiotic stresses and the plant responses to those stresses are somehow related to one or several structural, physiological, biochemical and/or molecular mechanisms pertaining to the plant tolerance. With this review, we have gained sufficient understanding of basic and various underlying mechanisms that are involved in plant stress tolerance through using exogenous Ca^2+^, however, more and thorough researches should focus on its mitigating effect and its associated tolerant genes and use them in crop breeding for more resilient varieties and cultivars against extreme abiotic conditions. Also, all abiotic stresses are variables and their impact on plant growth are different and difficult to predict, so plants would adjust themselves constantly to adapt those fluctuations, indicating the quantity, method and timing of using different type of exogenous Ca^2+^ can be critically important in maximizing the Ca^2+^ efficacy.

## Mechanisms of the role of exogenous Ca^2+^ in plant resistance against abiotic stresses

### Stabilization of cell wall and membranes

Stresses imposed on plants such as salinity, high temperature, low temperature, and drought tend to induce more reactive oxygen species (ROS) that cause a peroxidation of cell walls and membranes and change the membrane permeability, resulting in an osmotic disturbance. While being applied, exogenous Ca^2+^ mainly reduced the ion leakage (Min et al., 2021), replenished the lost Ca^2+^, induced the synthesis of osmoregulatory substances (Hu et al., 2012; Naeem et al., 2020), increased antioxidant enzyme activities such as superoxide dismutase (SOD), peroxidase (POD), and catalase (CAT) et al., (Upadhyaya et al., 2011), and promoted biosynthesis of glutathione (GSH), ascorbate, tocopherols, and other non-enzymatic antioxidants (Ashraf, 2009; Elkelish et al., 2019). All these biological and physiological responses, secondary metabolites and pertaining enzymes have proven to maintain the stability and integrity of plant cell walls and membranes. For example, exogenous Ca^2+^ would facilitate the accumulation of gamma-aminobutyric acid (GABA) and free polyamines (PAs) to alleviate cell membrane damage (Yin et al., 2015), mitigate the decline of unsaturated lipids incorporated in cell membranes through producing and supplying more unsaturated fatty acids as for retaining membrane fluidity (Liang et al., 2021), and combine with phosphate, organic phosphorus, and carboxyl groups of proteins on the cell surface to stabilize the cell membrane structure and maintain cell integrity (Hu et al., 2018).

### Regulation of the Na^+^/K^+^ ratio

Both Ca^2+^ and K^+^ are ions necessary in plants for the protein synthesis, antioxidant enzyme activity, and maintenance of plasma membranes and cell walls (Assaha et al., 2017) and it has been consented that the Na^+^/K^+^ ratio is mainly regulated in response to salt stress and the key factor for plants to tolerate salt stress is to keep the Na^+^/K^+^ ratio low (Shabala and Pottosin, 2014). Ca^2+^ incorporated in the plasma membrane in plants under a salt stress is replaced by a large amount of Na^+^ that are usually available due to an excessive NaCl influx, leading to an increase in cell membrane permeability and causing an intracellular K^+^ extravasation, a high Na^+^/K^+^ ratio, and an insalubrious ionic balance (Cabot et al., 2014). An administration of exogenous Ca^2+^ cannot only control the Na^+^ entry into plants through non-selective cation channel (NSCC), reduce K^+^ efflux or leakage through both NSCC and the guard cell outward rectifying potassium channels (GORK), but also increase antioxidant enzyme activities and quantity of osmoregulatory substances to reduce the ROS accumulation that opens NSCC for ion leakage and keeps the ion homeostasis balanced in stressed plants (Rahman et al., 2016). In addition, a recent research has indicated that the salt overly sensitive (SOS) pathway was initiated by the enhanced Ca^2+^ signals that were stimulated by exogenous Ca^2+^, which promoted a Na^+^ efflux and more K^+^ uptakes through the SOS pathway in wheat (Gao et al., 2021).

### Regulation of hormone levels in plants

Plant growth, development and reproduction are basically regulated by endogenous hormones and their fluctuations in plant can be influenced by and responded to changes of environment conditions (Mesejo et al., 2013). Ca^2+^ censors include calmodulins (CaMs), CaM-like proteins (CMLs), calcineurin B-like proteins (CBLs), and CDPKs. The Ca^2+^ sensor is an initial stress signal detector as well as a regulator of major plant hormone signals (Ku et al., 2018). Ca^2+^ is reportedly involved in the ABA-induced stomatal closure process (Liu H et al., 2022), with which ABA is participated in the initiation and release of Ca^2+^. In addition, both Ca^2+^ and ABA regulating kinases target the same metabolic pathway (Edel and Kudla, 2016) through regulating the biosynthesis and signal transmission of jasmonates (JAs) that subsequently adjust the Ca^2+^ level, inducing an influx of extracellular Ca^2+^, and temporarily increase its concentration in the nucleo plasma. So, Ca^2+^ signal is regarded as the most important messenger in the signal cascade (Wang X et al., 2019). Ca^2+^ can control the transport rate of Indole-3-acetic acid (IAA) and switch the direction of IAA flow to effectively amply Ca^2+^ signaling for activation of cation pumps in the plasma membrane, promotion of Ca^2+^ influx and K^+^ efflux, and induction of root gravity by interacting with IAA (Vanneste and Friml, 2013). However, the Ca^2+^ molecular basis of mechanisms involved in the signaling, Jas pathway, IAA biosynthesis are still poorly understood. Under adverse conditions, exogenous Ca^2+^ proved to alleviate potentially damaging effects caused by the stress on plant growth and development through minimizing the ABA amount and increasing the production of other hormones (*e.g.*, IAA, GA, CTK, *etc.*) to enhance plant resilience under stress (Liu et al., 2017; Kamran et al., 2021; Wu et al., 2022). At present, fewer studies of exogenous calcium on its effect on changes of a variety of plant hormones have been reported but mainly focused on ABA fluctuations and their associated impacts.

### Maintenance of photosynthesis

Plants needs the chlorophyll as an life-supporting pigment for photosynthesis, and its quantity in plant leaves directly affects the photosynthetic capability to produce carbohydrates (Wu et al., 2019). Adversary stresses tend to impair the chloroplasts and cause a decrease in the chlorophyll quantity. Exogenous Ca^2+^ could prevent or minimize chlorophyll breakdowns, keep chloroplasts intact under stresses, and maintains a sufficient number of photosynthesis pertaining pigments and organelles in in leaves (Min et al., 2021; Wang et al., 2022). Ca^2+^ plays an important role in plant stomatal regulation as the second messenger in coupled with external signals in plant cells. Appropriate amount of Ca^2+^ can make plants adapt to abiotic stresses such as drought and salinity quickly by adjusting their stomatal opening/closing, optimizing their gas exchange, and improving their photosynthetic efficiency (Li et al., 2012; Li et al., 2022a). These plant adjustments and adaptations by using exogenous Ca^2+^ could be achieved through reshape, rearrangement and configuration of stomata during their differentiation and development for more efficient of gas exchange and water utilization (Zhang et al., 2019a). Moreover, adding exogenous Ca^2+^ to increase Ca^2+^ level improved the lutein cycle (Yang et al., 2013), mitigated the adversary effects on the photosystem II (PSII) inhibition, and preserved enzyme activities, reduced accumulations of carbohydrate, and uphold a normal plant photosynthesis (Tan et al., 2011).

### Regulation of plant respiratory metabolism and improvement of root activities

Flooding causes anoxia of plant roots and weakens the respiratory metabolism. The stress due to flooding-initiated lack of oxygen can be lessened by the application of exogenous Ca^2+^ to improve the catalytic capacity of pentose phosphate and produce more ATP and NADPH to provide more energy for the plant respiratory metabolism (Yang et al., 2016), to promote the activity and accumulation of mitochondrial antioxidant enzymes relating to the glycolysis and tricarboxylic acid cycle (He et al., 2015), and to reduce the content of acetic acid, acetaldehyde and the activity of LDH for less lactic acid metabolism (Fan, 2019). Exogenous Ca^2+^ also proved to facilitate absorption of nitrate and accelerate its conversion into amino acids, heat stable proteins or polyamines to survive from hypoxia (Gao et al., 2011). In addition, some studies on plant salt stress also pointed out that exogenous Ca^2+^ can improve the root vitality by reducing the relative electrolyte leakage of the root, thus to improve the flooding tolerance of foxtail millets (Han et al., 2019). According to the summary of current literature, this mechanism mainly plays an active role under flooding stress, and whether it can also be activated under other stresses is unclear.

### Induction of gene expressions and protein transcriptions for the stress resistance

While under the abiotic stress, the molecular mechanisms involved in plant stress tolerance are more complex and multi-layered, including stress sensing, responsive signaling, gene transcription, protein translation, and post-translational protein modification. Under various abiotic stresses, exogenous Ca^2+^ induces or activates a series of gene expressions and tolerant protein transcriptions to adjust and adapt to adversities accordingly. These research advances in that regards include but are not limited on: 1) upregulating the expression of antioxidant enzyme-related genes such as *EnAPX*, *EnCAT2*, *EnGPX* and stress-related genes to improve cold resistance of *Elymus nutans* (La-mu et al. 2021); 2) promoting the synthesis of plant proteins and preventing proteins from degradation through boosted activities of nucleoside diphosphate kinase (NDPK) and antioxidant enzymes and reduced expressions of heavy metal-related structural domain proteins such as *PCR1*, *HMA2* and *HMA4l* (Zeng et al., 2017); 3) cutting down the Cd uptake of plants and promoting the Ca^2+^ internal mobility (Zeng et al., 2017); 4) stimulating *ACO-1*, *ADH-1*, *CAT-2*, and *PK* gene expression to alleviate the damage of pepper plants under flooding stress (Ou et al., 2017), and 5) inducing the expression of photosynthetic genes and stabilizing photosynthetic membrane proteins in leaves (Zhang et al., 2014). Due to the differences of gene pools in different plant species and the difficulties in monitoring those genes, the information we currently have on plant genomes is still limited, focusing only on detecting gene expressions and reflecting Ca^2+^ associated genetic changes after application of exogenous Ca^2+^, but how exactly these adjustments are induced, operated and regulated remain to be explored. In addition, how exogenous Ca^2+^ transduces Ca^2+^ signaling pathway in plants has not been determined yet.

## Summary and outlook

**Figure 2:**
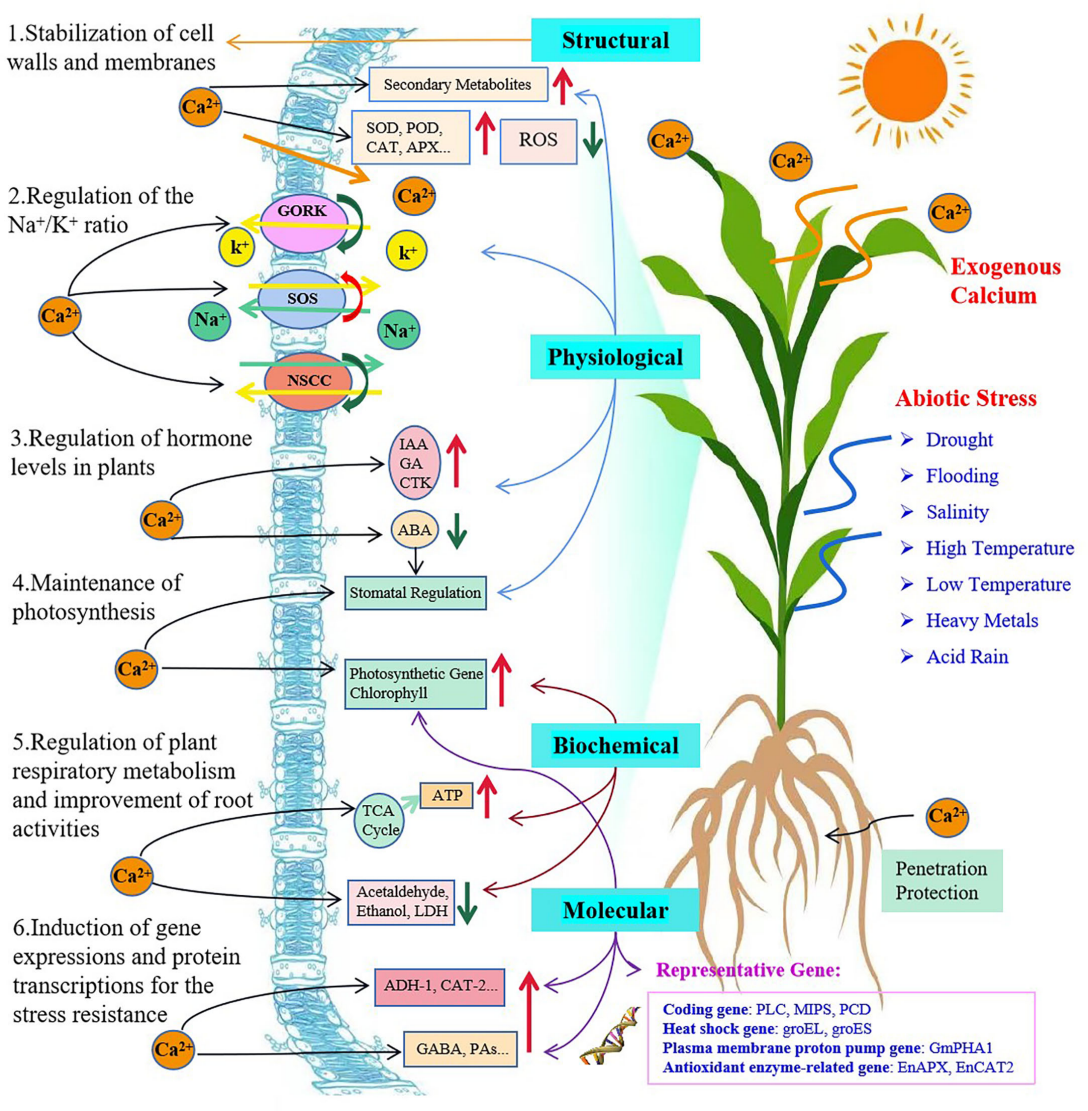
Plant self-responses to abiotic stresses and mechanisms of exogenous calcium involved in enhancement of plant stress tolerance. The red and green arrows indicate a promotion/increase or an inhibition/decrease, respectively.

With an extensive review of over one hundred research papers, we have sorted them according to their major mechanisms into six categories associated with the plant membranes, Na^+^ vs. K^+^ ratios, hormone regulation, gene expression and protein transcription, and photosynthesis. However, we tend to believe that this type of grouping is arbitrary, simplified and nonscientific only for the purpose of easy access and preliminary understanding of a particular aspect of main functions of exogenous Ca^2+^ that may have alleviated a certain type of plant stresses. The mechanisms involved in mitigating plant abiotic stresses through application of exogenous Ca^2+^ and their reported interrelationships are proposed and demonstrated (**Figure 2**) and we strongly suggest that they be perfected and completed with more fundamental information and advanced findings are available.

In the process of reviewing all relevant literature, we have found that most of the studies only explored one or a few aspects of stressed plants in response to exogenous Ca^2+^ added to alleviate a stress, but the application of exogenous Ca^2+^ may have a potential to affect multiple structural, biological and physiological functions or metabolic pathways at the plant cellular level to defend plants from various stresses. Likewise, exogenous Ca^2+^ could be used with other exogenous substances to enhance plant defensiveness against one or multiple stresses that are related or associated with each other to intensify the adversary impact, such as the stress from drought, high temperature and high salinity since they are somehow correlated. Other studies have shown that exogenous Ca^2+^ is more effective when combined with other exogenous substances, which should be our research directions and objectives for our endeavor in using exogenous Ca^2+^ in the near future (Vafadar et al., 2020; Valivand and Amooaghaie, 2021). Therefore, more and more well-designed experiments to unearth the true underline mechanisms of exogenous Ca^2+^ in mitigating multiply correlated plant abiotic stresses are expected and the results derived from them should significantly help us understand how to effectively use of exogenous Ca^2+^.

It has come to a consensus that exogenous Ca^2+^ can be used to alleviate various abiotic stresses on plants through an application of leaf spray, hydroponics, seed dipping, drenching, and soil application. To avoid possible interference of NO_3_^-^ and other nutrient anions to the experimental results, most of the existing studies on exogenous Ca^2+^ used CaCl_2_ as the source calcium for a foliage spraying or through hydroponics. However, with the studies of the heavy metal stress, exogenous Ca^2+^ such as Ca(OH)_2_ was mostly used in the form of mixing it into the medium or soil (Zu et al., 2020) to increase soil pH, reduced the toxicity of heavy metals, and boost the Ca^2+^ quantity both in soil and plants. In addition, different calcium anions also affect plant growth. For example, the application of exogenous CaCl_2_ on chloride-phobic cowpea plants under stress could cause the accumulation of Cl^-^ in roots and affect the normal growth (Guimarães et al., 2011), which in turn interferes with a positive mitigating effect of Ca^2+^. Therefore, the research in the future should be needed as well to investigate the type of exogenous Ca^2+^ that is suitable for the growth and development of a specific crop with an attention to determine the amount of the usage based on the type and extent of adversary stresses.

At present, majority of the experimental studies have been carried out indoors and mainly on the plant seed germination or seedlings, however, we believe that the best way to evaluate the effect of exogenous Ca^2+^ for a practical and feasible application should be carried out in an actual crop production site to determine its mode of action, actual concentration and optimal time for application, *etc*., or if priming of plant seedlings for their fortified and prolonged tolerance against abiotic stresses really words in field trials.

In summary of most recent advances on exogenous Ca^2+^ applications to alleviate various plant stresses, some questions still remain unanswered in terms of: 1) how Ca^2+^ is further transported and translocated after it enters a plant; 2) how efficiently Ca^2+^ is actually utilized to function as a mitigating factor; 3) in which way the Ca^2+^mobility can be improved; 4) how to use modern molecular assays to reveal detailed and Ca^2+^-induced mechanisms pertaining to the plant tolerance against abiotic stresses; 5) what are the interactions between different biological and physiological mechanisms that are all modulated by gene expressions and protein transcriptions at the molecular level; and 6) to what degree each of the abiotic stresses causes an irreversible and permanent damage. To address those challenging questions, the modern molecular techniques and more sophisticated analytic instruments such as an fluorescence tracing technique and a laser scanning confocal microscopy analysis technique (He et al., 2015) should be used for a quantitatively and qualitatively detection of a series of changes in signaling and gene expression induced by exogenous Ca^2+^. With this review, we have sorted a series of complex physiological and biochemical responses and their underline mechanisms that were reported recently, but much more deserve further exploration by researchers to develop a low-cost and effective way to combat all kinds of stresses though using exogenous Ca^2+^.

## Author contributions

Conceptualization: DF. References analysis: XW. Funding acquisition: DF. Methodology: DF, XW. Validation: DF, XS. Writing–original draft: XW, DF, JG. Writing–review & editing: XS, CZ, HL, PL. All authors contributed to the article and approved the submitted version.
